# Relationship between Psychological Capital and Psychological Well-Being of Direct Support Staff of Specialist Autism Services. The Mediator Role of Burnout

**DOI:** 10.3389/fpsyg.2017.02277

**Published:** 2017-12-22

**Authors:** Guadalupe Manzano-García, Juan-Carlos Ayala

**Affiliations:** ^1^Department of Sciences Education, University of La Rioja, Logroño, Spain; ^2^Department of Economics and Business, University of La Rioja, Logroño, Spain

**Keywords:** psychological well-being, psychological capital, burnout, direct support staff, autism services

## Abstract

This study investigates the specific role of burnout as a mediator in the relationship between psychological capital and psychological well-being (PWB) in direct support staff of specialist autism services. A time lagged design with three data-collection points was conducted to survey 56 professionals (direct support staff) who work at a Spanish center specialized in autism. Participants completed measures of psychological capital, burnout and PWB. The hypothesized model was tested using structural equation modeling. Our findings show that psychological capital has a significant main effect on PWB. The results also show that psychological capital in the work environment should result in lower burnout which in turn, should lead to higher degrees of PWB in the direct support staff of autism services. Our results support that psychological capital is a key variable in the working life of the direct support staff of autism services. The findings suggest the need of implementing programmes which strengthen each individual's psychological capital in order to prevent burnout and achieve a greater PWB.

## Introduction

People affected by Autism Spectrum Disorder (ASD) have a unique set of characteristics that differentiate them from others. People with ASD have problems with language usage and understanding, and have difficulties relating to people, things and events. They experience limitations with social relationships (e.g., trouble making friends) and when interpreting the facial expressions of others, which leads them to avoid eye contact. They also have difficulties adapting to unfamiliar environments and when changes occur in their daily routine (Bonis, 2016). There are differences in their sensory perception. They experience the world differently. In short, they perceive things in their environment differently because they process information differently from most people. They may have problems amalgamating and integrating information received through their senses, affecting perception, understanding and behavior (American Psychiatric Association, 2013). They often fall victim to criticism and bullying and respond with isolation, aggression, hostility, or self-injury (Horner et al., 2002).

The characteristics of people who have ASD and the demands associated with their care mean that their caregivers face constant challenges (Lovell et al., 2014; Lovell and Wetherell, 2016). The difficulties faced by these caregivers are exacerbated when their psychological capital is low, which is often associated with increased emotional exhaustion and decreased psychological well-being (PWB). Previous literature, using samples of caretakers (normally parents) of people affected by ASD, has explored the influence of psychological capital on PWB (Newman et al., 2014). However, there are virtually non-existent investigations exploring these relations in the direct support staff of specialist autism services. Previous research has also shown that caregivers of people affected by ASD experience high levels of burnout. Lovell and Wetherell (2016) and Laschinger and Grau (2012) have suggested that burnout may moderate the relationship between psychological capital and PWB. Nevertheless, this hypothesis has never previously been tested in direct support staff of autism services. Thus, our research had two objectives: (a) to examine the direct effect of psychological capital over the PWB of the direct support staff of specialist autism services and, (b) to explore if burnout is a significant mediator in the relationship between psychological capital and PWB of direct support staff of specialist autism services. Knowing how psychological capital and burnout affect PWB levels of direct support staff of autism services could be important for: (a) keeping their physical and mental health, which deteriorates with burnout; (b) to provide valuable information in the development of individual and organizational intervention strategies.

## Theoretical background

### Psychological well-being and psychological capital

Psychological well-being (PWB) is a derivative of the positive psychology field and entails the cultivating of positive emotions to ensure the optimal functioning and experience of individuals (Ryan and Deci, 2001). During the last decade there has been much research into PWB (Díaz and Sánchez, 2002) that has explored the concept map on this construct. Ryan and Deci (2001) suggested two large blocks of studies: those related to happiness (hedonic well-being), and those linked to human potential (eudaimonic well-being). The hedonic tradition conceived well-being to be an indicator of the quality of life between environmental characteristics and the degree of satisfaction felt by people (Campbell et al., 1976). It was subsequently defined as life satisfaction: a general judgment people make of their lives (Cabañero et al., 2004), or in terms of happiness. Furthermore, the eudaimonic aspect is more cognition-based and focused on the motivation individuals possess to achieve their goals and thereby contributes to positive feelings (Culbertson et al., 2010).

Psychological well-being (PWB) focuses on the development of skills and personal growth. Its origins are structured around concepts such as self-actualization (Maslow, 1968), full functionality (Rogers, 1961) or maturity (Allport, 1961). Ryff (1989) proposed a PWB multidimensional model consisting of six dimensions: self-acceptance, positive relationships with others, autonomy, environmental mastery, purpose in life, and personal growth. One of the central criteria of PWB is self-acceptance (SA). Despite each human being's limitations, people try to feel good about themselves (Keyes et al., 2002). The ability to positively relate (PR) to others and the ability to love are fundamental characteristics of a positive psychological activity and good mental health (Keyes et al., 2002). Another essential dimension of PWB is autonomy (A). A person must maintain their own convictions, personal authority and independence in different social contexts in order to strengthen their own individuality (Ryff and Keyes, 1995). Autonomy leads people to better withstand social pressure and control their own behavior better (Ryff and Singer, 2002). Environmental mastery (EM) refers to the individual's ability to create or choose environments that meet their personal needs and desires. Individuals who have environmental mastery have a sense of greater control over the world around them and are able to influence their context. Furthermore, in order to achieve PWB, human beings need goals that give meaning to their lives; they need to have a life purpose (LP). Additionally, they must persevere in developing their potential and capacity to continue developing and growing as individuals (Keyes et al., 2002). It is a dimension known as personal growth (PG).

The psychological capital (PC) construct refers to individuals who positively value daily life events and increase their chances of success by relying on perseverance and effort. It is a positive state of individual psychological development characterized by (Luthans et al., 2007) self-efficacy, optimism, hope and resilience. Self-efficacy (SE) is a beliefs in “people's judgments of their capabilities to organize and execute courses of action required to attain designated types of performances” (Bandura, 1997). Self-efficacy beliefs determine our behavior, feelings and thoughts. These capabilities specify the effort to overcome obstacles and perseverance in trying to achieve something. Optimism (O) can be analyzed from two theoretical perspectives: Peterson and Seligman's optimistic-pessimistic explanatory style (Peterson and Seligman, 1984), and Scheier and Carver's dispositional optimism (Scheier and Carver, 1987). This study is based on Peterson and Seligman's (1984) optimistic-pessimistic explanatory style. Seligman (1998) defines optimism as a cognitive process, the expectation of positive results and their corresponding functions. Hope (H) is a positive motivational state that has the energy and ability to organize and redirect, if necessary, in order to obtain objectives or goals (Snyder, 2002). People who have hope are more likely to achieve their goals by focusing more on successes than failures; they have fewer negative emotions when faced with obstacles to achieve their goals because of their ability and willingness to resort to different options (Youssef and Luthans, 2007). For Bonanno (2004), resilience (R) is the ability to overcome adversity, failure and conflict, and the ability to recover and come out stronger after having been subjected to highly stressful or extremely positive events.

**Figure 1:**
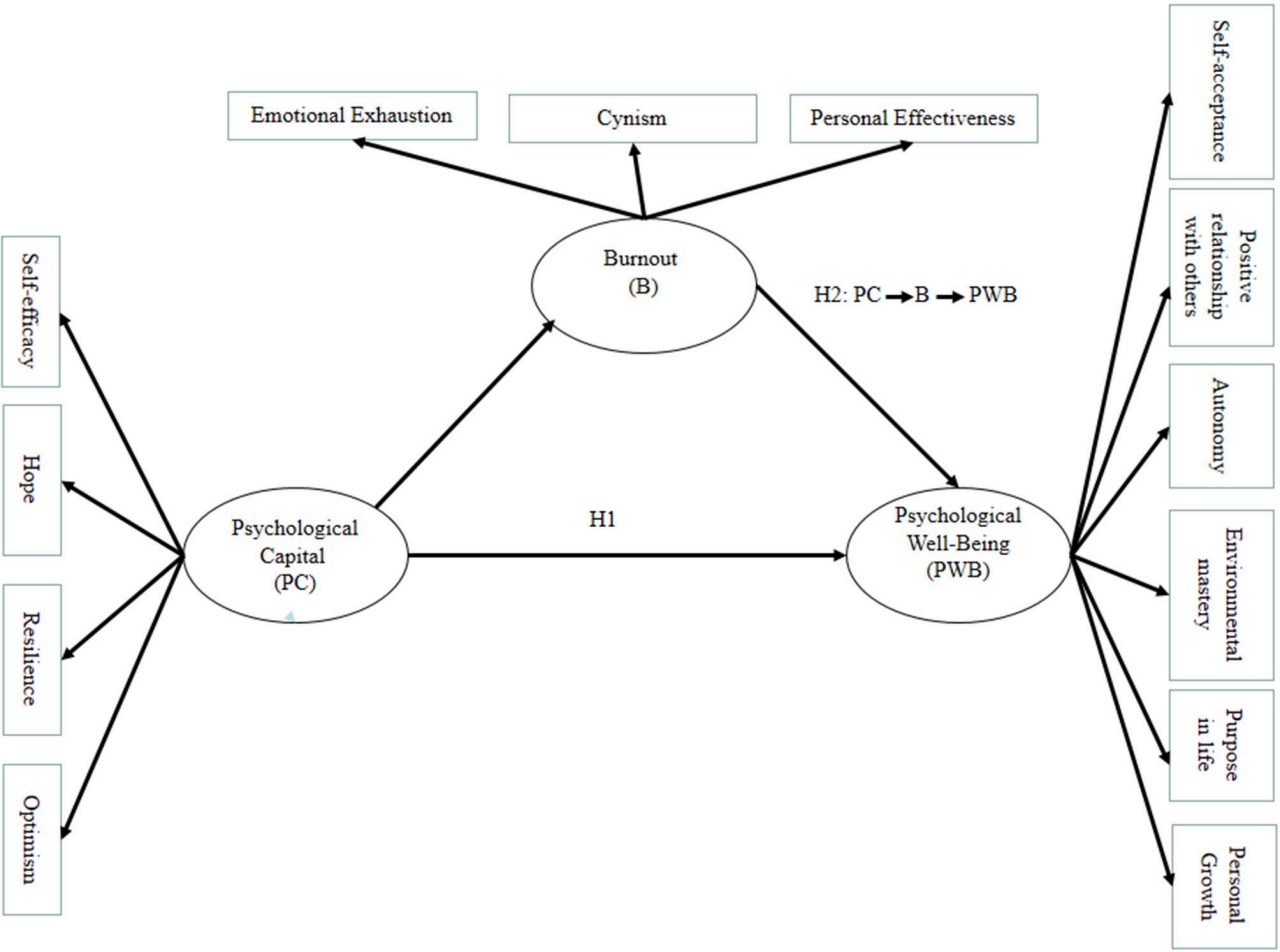
Research model.

The definition of psychological capital (PC) emphasizes the fact that these positive psychological skills have properties that can be improved. Psychological capital is an open construct that has development capacity which means it plays an important role in individuals' improvement (Luthans and Avolio, 2003). Psychological capital helps to trigger cognitive, affective, conative and social mechanisms, leading to PWB (Avey et al., 2010; Newman et al., 2014); it can facilitate the attention, interpretation and memory retention processes necessary for domain-specific experiences and satisfaction to render a lasting impact on PWB (Diener and Biswas-Diener, 2008). According to Sweetman and Luthans (2010), psychological capital is a personal resource that increases an individual's ability to handle difficult situations and personal pro-activeness, which promotes PWB and a suitable work performance. Hansen et al. (2015) also found a positive relationship between psychological capital and PWB. Luthans et al. (2007) showed that PWB can be predicted better when the dimensions of psychological capital are considered as a whole (a multidimensional construct) rather than considering the individual resources independently. In line with these findings, we hypothesize (Figure 1):

Hypothesis 1: Psychological capital of the direct support staff of specialist autism services is positively related to PWB.

### Psychological well-being, psychological capital and burnout

Direct support staff of specialist autism services work with people with a different progression of the ASD. In some occasions, these professionals find themselves exposed to the self-damaging and violent behaviors of the people they offer their services to. These behaviors tend to be related with the level of difficulty in communication and the mobility of the people affected by ASD (Collacott et al., 1998), as well as with the break of daily routines which they need to preserve in order to make their daily life more foreseeable and easy to understand. The work of the direct support staff is centered upon reducing the demands of the person they offer their services to as well as on trying not to block any of his/her self-stimulating behaviors, as may be the flapping of hands. Moreover, s/he has to work without intruding upon the personal space of the person s/he takes care of and to avoid, as much as possible, loud noises and disorder. Added to that, s/he must get used to using a clear and simple language, with the use of pauses that will facilitate the interlocutor to process language (Scarpinato et al., 2010). Moreover, s/he has to do the job avoiding, as far as possible, visual contact as this tends to be stressful for people with ASD (Browne, 2006). In such a structured and programmed context, the work of the direct support staff of autism services tends to be oriented solely to task doing, thus reducing the verbal interaction with the people they offer their care to. This favors the appearance of such feelings as lack of freedom, loneliness, apathy, incomprehension, etc. (Franz et al., 2010). This may lead the direct support staff of specialist autism services to burn out, which may negatively affect their PWB, their quality of life (Tung et al., 2014) and the quality of the services provided (Dewey et al., 2007).

Burnout it is a three-dimensional syndrome characterized by emotional exhaustion, cynicism and a sense of inefficiency and lack of achievement at work (low self-efficacy) (Maslach, 2009). Emotional exhaustion (EE) is the core of the syndrome. It refers to a feeling of lacking the emotional and physical resources needed to respond to the demands of care seekers. Cynicism (C) represents a component of the interpersonal context of burnout and refers to a negative, insensitive or excessively apathetic response to different work factors. Low personal effectiveness (PE) represents the self-evaluation component of burnout and refers to feelings of incompetence and lack of achievement and productivity at work. Burnout (B) is related to the work and the role that the person plays in their work (Lim et al., 2010). Researchers seem to agree that factors related to work are key in the development of burnout (Pines and Keinan, 2005). Burnout is especially prevalent among workers who are in permanent contact with people (Maslach and Leiter, 2016). In the case of direct support staff of autism services, usual stressful factors (work overload, conflicts and ambiguities of role, etc.) it must be added that professionals are trapped between the expectations and needs of the parents and the people they offer their services to, the behavioral problems shown by the people they take care of, the excessively structured and programmed character of the tasks to be done or the complex relationships with parents (Burrows, 2010), who think they are low-skilled to understand and tackle the challenges associated with the care of a person with ASD (Silva and Schalock, 2012).

Previous studies have shown that direct support staff of autism services have higher levels of perceived stress (Lovell et al., 2014). Professionals working with people who have ASD face complex situations that may lead them to feel insecure and powerless to control the stressful factors that caring for these people involves, and feel emotionally drained. Lovell and Wetherell (2016) found that direct support staff of autism services are prone to burnout. Luthans et al. (2004) support that employees with a high psychological capital are more likely to interpret positively the conditions of the working environment, and demonstrate a higher capacity of adaptation to the organizational climate and the changes that may occur in their working environment. Thus, it is logical to expect that the psychological capital could be related to burnout. Although very few studies have focused on direct support staff of autism services, previous research with other prone-to-burnout groups has shown that psychological capital may contribute to lower levels of burnout. For example, Friedman (1999) suggests that an increased professional self-efficacy could decrease emotional exhaustion. Wandeler and Bundick (2011) found a positive relationship between hope and perceived competence. Manzano-García and Ayala Calvo (2013) showed that resilient individuals have lower levels of burnout. Llorens et al. (2007) associated self-efficacy with vigor and dedication, two dimensions of engagement, as opposed to burnout. Vazi et al. (2011) demonstrated that high levels of psychological capital are associated to low levels of burnout. The psychological resources inherent in psychological capital (self-efficacy, hope, optimism, and resilience) may fight against the development and progression of burnout (Gökhan Bitmişa and Ergenelib, 2015). Furthermore, previous research, using different samples, also showed that high levels of burnout lead to a deterioration in PWB and a low quality of the care being provided. For example, Scanlan and Still (2013), Lizano and Mor (2015), and Harris et al. (2016), in samples of occupational therapists, educators and public child welfare workers, respectively, showed that the dimensions of burnout are useful in understanding PWB and that interventions that help manage burnout promote PWB. Laschinger and Grau (2012) using a sample of nurses showed that increased psychological capital was negatively related to emotional exhaustion, and it was a key variable which may contribute to decreased burnout and increased PWB. Having considered the statements expressed above, we hypothesize that (Figure 1):

Hypothesis 2: The relationship between psychological capital and PWB of the direct support staff of specialist autism services is mediated by burnout.

## Methods

### Ethics statement

This work follows the rules and procedures approved by the center's management committee which all participants in the study belong to. All procedures followed were in accordance with the ethical standards of the responsible committee on human experimentation (institutional and national) and with the Helsinki Declaration of 1975, as revised in 2000. The management committee reviewed and approved the ethical guidelines of research before the study began. Informed consent was obtained from all participants for being included in the study.

### Participants and procedures

The study sampled 56 direct support staff who work at Riojan Association of Parents of Children with Autism (Leo Kanner center). This is the only center in La Rioja region, Spain, that is dedicated to providing professional support to people affected by ASD. The main functions of the professionals who have participated in the study are: direct assistance and educational care of the centre's users; implementation and evaluation of the individual programmes of intervention; design of the educational activities; permanent evaluation of users; coordination with the remaining team and continuing communication with parents.

For the purpose of this study, all direct support staff who work at Leo Kanner center (*N* = 72) were requested to participate. They received a letter from the researchers containing a description of the study aims. They were also informed that the research was neither directed nor driven by the organization in which they worked and that their responses would be treated in the strictest confidence. All participants were confidential and volunteers, and 80% completed the questionnaires.

Consenting participants were surveyed on three different occasions in 2016. In the first half of March (T1), we gathered data on psychological capital; in the second half of March (T2), we gathered data on burnout; and in the second week of April (T3), we gathered data on PWB. In T1, T2 and T3 the questionnaire was enclosed, together with a return envelope. In T1 sent 72 questionnaires. Sixteen of direct support staff decided not to participate in the study and provided no feedback. The 56 responders who participated in T1 also participated in T2 and T3.

Most of the people in the sample were women (82.14%), single or divorced people (55.4%), working 40 h per week or less (89.29%) and having a permanent employment contract (76.79%). Direct support staff of autism services, on average, were 37.59 years old (*SD* = 10.53, range: 21–59), had 4.84 years' experience in their current workplace (*SD* = 3.42; range: 0.1–16) and 0.74 children (range 0–3).

### Instruments

#### Demographic questions

The first section of the questionnaire contained 7 items on socio-demographic data: age, gender (1 = male, 2 = female), marital status (1 = married or common-law partner; 2 = divorced or single), contract type (1 = permanent; 2 = temporary), experience in their current job, hours they work per week and number of children.

#### Psychological capital

Psychological capital was measured using the Spanish version (Azanza et al., 2014) of the psychological capital questionnaire (PCQ) developed by Luthans et al. (2007). The questionnaire measured self-efficacy (6 items, e.g., “I feel confident in analyzing a long-term problem to find a solution”), hope (6 items, e.g., “I have several ways to accomplish the work goal”), optimism (6 items, e.g., “I believe that all the problems occurring at work always have a bright side”) and resilience (6 items, e.g., “I usually manage difficulties one way or another at work”). Participants used a Likert-type response scale from 1 (Strongly disagree) to 6 (Strongly agree) to assess each item. Azanza et al. (2014) found adequate reliability. In our study, self-efficacy reliability was 0.70. For hope, resilience and optimism, Cronbach's alpha was 0.80, 0.70 and 0.74 respectively.

#### Burnout

Burnout was measured using the Spanish version of MBI-GS (Schaufeli et al., 1996; Salanova et al., 2000). The questionnaire consists of 15 items distributed in three dimensions: personal efficacy (6 items, e.g., “I think I have enough confidence in my efficacy to achieve my objectives”), emotional exhaustion (5 items, e.g., “Working all day is stressful for me”) and cynicism (4 items, e.g., “I have lost interest in my work since I started in this position”). Items were rated by participants by using a Likert-type scale ranging from zero “0” (never) to “6” (always). High scores on the emotional exhaustion and cynicism dimensions coupled with low scores on the personal efficacy dimension are indicative of burnout. Cronbach's alpha values found by Salanova et al. (2000) found adequate reliability. In our study, Cronbach's alpha values were: emotional exhaustion (α = 0.90); cynicism (α = 0.81); and personal efficacy (α = 0.72).

#### Psychological well-being

Psychological well-being (PWB) was measured using Van Dierendonck's PWB (Van Dierendonck, 2004) adapted into Spanish by Díaz et al. (2006). The instrument has a total of 29 items distributed into six scales: self-acceptance (4 items, e.g., “When I look at the story of my life I am pleased with how things have turned out so far”); positive relationships (5 items, e.g., “I feel that my friendships are beneficial for me”); autonomy (6 items, e.g., “I have confidence in my own opinions even if they are different from the way most other people think”); environmental mastery (5 items, e.g., “I've been able to create a home and lifestyle to my liking”); life purpose (5 items, e.g., “I enjoy making plans for the future and working to achieve them”); and personal growth (4 items, e.g., “Generally, over time, I feel as though I learn more about myself”). Participants rated each item by using a response format with scores that ranged from 1 (strongly disagree) to 6 (strongly agree). Díaz et al. (2006) found adequate reliability. In our study, Cronbach's alpha values were: self-acceptance (α = 0.83); positive relationships (α = 0.73); autonomy (α = 0.71); environmental mastery (α = 0.67); personal growth (α = 0.72) and purpose of life (α = 0.80).

### Data analysis

Prior to testing the hypothesized model, we first conducted a *t*-test in order to check whether psychological capital, burnout and PWB differed according to gender, marital status or type of contract. The results showed that on average, the PWB of people living with a partner is significantly higher than that of those living alone [*F*_(53)_ = 6.588; *p* = 0.013]. We then correlated age, years in current work, weekly work hours and number of children with psychological capital, burnout and PWB. The results showed that there were no significant correlations. Consequently, we decided to retain only marital status (control variable) in further analyses in order to achieve the maximum power for the following tests (Edwards, 2008). Statistical analyses were conducted using SPSS version 22.

The hypothesized path model described in Figure 1 was tested using partial least squares (PLS), a variance-based structural equation modeling (SEM). We used SmartPLS path modeling software (Ringle et al., 2015). The use of PLS-SEM (Partial Least Square-structural equation modeling) is justified for the following reasons: (1) this study is oriented toward the prediction of the dependent variables (Chin, 2010); (2) PLS is suitable for small samples (Reinartz et al., 2009); (3) the hypothesized model is complex: it includes constructs of the first and second order, and direct and mediated effects appear in the hypothesized relationships; (4) this study uses latent variables scores in the subsequent analysis for a predictive relevance (Hair et al., 2011).

PLS-SEM models were analyzed and interpreted in two steps (Aldás, 2015): (1) *Validating the measurement (outer) model*. In this stage, the PLS-SEM is estimated including its structural (inner) part, but no attention is paid to the estimation of the regression coefficients. Only the weights and loadings of the outer model are considered and classical reliability and validity criteria will be applied. An item's individual reliability is assessed by examining the loading factors (λ), or the simple correlations of the measurements or indicators with their respective construct. In general, to accept an indicator as being integral to a construct, it must have a load equal to or superior to 0.7. However, following Hair et al. (2014) criterion, we removed the items with an individual reliability above 0.4 and below 0.7 but only if their removal had a significant influence on the rise of the Media Extracted Variance (AVE) of the construct. The items presenting an individual reliability below 0.4 were eliminated. The reliability of a construct analyses internal consistency for a given group of indicators and is measured using composite reliability (ρ_c_) as an indicator. The usual criterion is that (ρ_c_) should be higher than 0.7 (Nunnally and Bernstein, 1994). The convergent validity of each constructs, is measured by Average Variance Extracted (AVE), and its value should be above 0.5 (Fornell and Larcker, 1981). Discriminant validity indicates the extent to which a given construct is different from other constructs. For discriminant validity to exist, Average Variance Extracted (AVE) must be greater than the variance shared between the construct and other constructs in the model (the correlation with the square between two constructs); (2) *Assessing the structural (inner) model*. Before interpreting the model results in terms of acceptance or rejection of our model hypotheses that we transformed into paths in the inner model, we need some guidance on the “quality” of the estimations it provides. Two criteria are commonly used: the R^2^ of the dependent construct and the predictive relevance. The statistical significance of the relations considered in the path model was evaluated according the R^2^ of the dependent construct and the standardized path coefficients, ß. Bootstrapping (5,000 samples) was used to generate standard errors and *t*-values of the parameters. Power analysis was developed with G^*^Power (Faul et al., 2007) which provide a statistical power for R^2^ deviation from zero. Higher than the 80 per cent is the level recommended. In order to demonstrate the predictive significance of the model we calculate Q^2^ statistics tests obtained by blindfolding (Geisser, 1975). Values of Q^2^ higher than zero demonstrate the predictive significance of the model.

To determine what role psychological capital (PC) plays, together with burnout (B) in the prediction of the PWB level of direct support staff, we proceeded in two steps. In each one of these, a new relationship (structural path) was added to that specified in the previous step. In the first step (Model 1), we measured the total effect of PC on PWB (H1). In the second (Model 2), we introduced B as a mediator in the relationship between PC and PWB (H2). Our purpose was to find out what part from total effect of PC on PWB is produced due to the direct effect of PC on PWB and what part is produced due to the indirect effect of PC on PWB through B. All constructs were designed as multidimensional constructs. To operationalize the multidimensional constructs, we followed a two-step approach (Faul et al., 2007). In the first step, using the PLS algorithm in a model without second-order constructs, the scores for each of the first order constructs were estimated. In the second step, we used these scores as observed indicators of the second-order constructs.

## Results

Table 1 shows the means, standard deviations, and correlations of the main variables of the study.

**Table 1:** Means, standard deviations and correlations of the main variables.

	**Mean**	**SD**	**1**	**2**	**3**	**4**	**5**	**6**	**7**	**8**	**9**	**10**	**11**	**12**	**13**	**14**
1. Resilience	18.80	2.98	(0.70)													
2. Optimism	17.84	3.30	0.54^**^	(0.74)												
3. Hope	22.89	4.33	0.49^**^	0.56^**^	(0.80)											
4. Self-efficacy	19.47	2.65	0.35^**^	0.31^*^	0.24^*^	(0.70)										
5.Emotional exhaustion	10.29	6.07	−0.44^**^	−0.65^**^	−0.54^**^	−0.21	(0.90)									
6. Cynicism	4.94	4.44	−0.33^*^	−0.61^**^	−0.57^**^	−0.17	0.72^**^	(0.81)								
7. Personal efficacy	19.57	3.14	0.13	−0.02	0.25^*^	0.30^*^	−0.25^*^	−0.31^*^	(0.72)							
8. Environmental mastery	15.14	2.35	0.25^*^	0.31^*^	0.23^*^	0.29^*^	−0.27^*^	−0.38^**^	0.27^*^	(0.67)						
9. Life purpose	24.43	4.04	0.36^**^	0.47^**^	0.69^**^	0.32^*^	−0.36^**^	−0.46^**^	0.29^*^	0.57^**^	(0.80)					
10. Personal growth	16.27	1.76	0.33^*^	0.45^**^	0.51^**^	0.27^*^	−0.28^*^	−0.36^**^	0.30^*^	0.49^**^	0.53^**^	(0.72)				
11. Autonomy	18.75	3.67	0.36^**^	0.34^*^	0.38^**^	0.35^*^	−0.19	−0.22	0.23^*^	0.27^*^	0.38^**^	0.23^*^	(0.71)			
12. Positive relationships	21.76	2.54	0.38^**^	0.37^**^	0.17	0.20	−0.18	−0.29^*^	0.25^*^	0.51^**^	0.37^**^	0.45^**^	0.31^*^	(0.73)		
13. Self-acceptance	20.44	2.80	0.35^*^	0.46^**^	0.46^**^	0.38^**^	−0.50^**^	−0.54^**^	0.28^*^	0.61^**^	0.63^**^	0.47^**^	0.43^**^	0.34^*^	(0.83)	
14. Marital status	1.55	0.50	0.00	−0.23^*^	−0.20	0.05	0.16	0.20	−0.05	−0.30^*^	−0.27^*^	−0.09	−0.30^*^	−0.04	−0.23^*^	–

*Cronbach's α reliabilities for the scales are shown in parentheses along the diagonal*.

** *Significant at p < 0.01*,

* *significant at p < 0.05*.

### Measurement model

Table 2 shows the main parameters corresponding to the measurement model: the individual reliability of the items as well as the compound reliability and the convergent validity of the constructs. The individual reliability of all items, assessed by standardized loadings, was sufficient (>0.5) (Barclay et al., 1995). The composite reliability of each of the constructs was greater than 0.7, which indicates that all the variables meet the requirement of construct reliability (Nunnally and Bernstein, 1994). The convergent validity of these latent variables, measured using the average variance extracted (AVE), was higher than 0.5 (Fornell and Larcker, 1981).

**Table 2:** Measurement model: loadings, construct reliability, and convergent validity.

**Construct/dimension/indicator**	**Loading**	**Composite reliability**	**AVE**
**Psychological capital**		**0.82**	**0.54**
Resilience (R)		0.81	0.52
R1	0.79		
R2	0.83		
R5	0.57		
R6	0.68		
Optimism (O)		0.84	0.57
O1	0.70		
O2	0.56		
O3	0.87		
O4	0.85		
Hope (H)		0.87	0.57
H1	0.62		
H2	0.74		
H3	0.60		
H4	0.90		
H5	0.88		
Self-efficacy (SE)		0.82	0.53
SE1	0.80		
SE2	0.80		
SE3	0.77		
SE4	0.51		
**Burnout**		**0.73**	**0.57**
Emotional exhaustion (EE)		0.92	0.71
EE1	0.91		
EE2	0.76		
EE3	0.89		
EE4	0.78		
EE5	0.85		
Cynicism (C)		0.88	0.64
C1	0.87		
C2	0.89		
C3	0.73		
C4	0.70		
Personal efficacy (PE)		0.82	0.53
PE1	0.68		
PE2	0.79		
PE3	0.73		
PE4	0.71		
**Psychological well-being**		**0.87**	**0.53**
Environmental mastery (EM)		0.82	0.60
EM1	0.79		
EM2	0.74		
EM3	0.79		
Life purpose (LP)		0.86	0.56
LP1	0.53		
LP2	0.81		
LP3	0.85		
LP4	0.79		
LP5	0.71		
Personal growth (PG)		0.80	0.57
PG1	0.74		
PG2	0.61		
PG3	0.90		
Autonomy (A)		0.82	0.53
A1	0.80		
A2	0.71		
A3	0.70		
Positive relationships (PR)		0.83	0.55
PR1	0.81		
PR2	0.79		
PR3	0.66		
PR4	0.69		
Self-acceptance (SA)		0.89	0.66
SA1	0.78		
SA2	0.83		
SA3	0.84		
SA4	0.80		
**Marital status**	**1**	**1**	**1**

Furthermore, as shown in Table 3, the square root of average variance extracted (the diagonal elements) are significantly greater than the correlations between the constructs (the off-diagonal elements in the corresponding rows and columns), thus allowing us to confirm the discriminant validity of the latent constructs in the pattern (Barclay et al., 1995). According to these results, the measurement model is correct.

**Table 3:** Measurement model: discriminant validity.

	**PC**	**B**	**PWB**	**MS**
1. Psychological capital (PC)	(0.73)			
2. Burnout (B)	−0.62^**^	(0.76)		
3. Psychological well-being (PWB)	0.61^**^	−0.63^**^	(0.73)	
4. Marital status (MS)	−0.22	0.21	−0.34^*^	(1)

*Square root of Average Variance Extracted (AVE) are presented in parentheses along the diagonal*.

** *significant at p < 0.01*,

* *significant at p < 0.05*.

### Structural model

**Figure 2:**
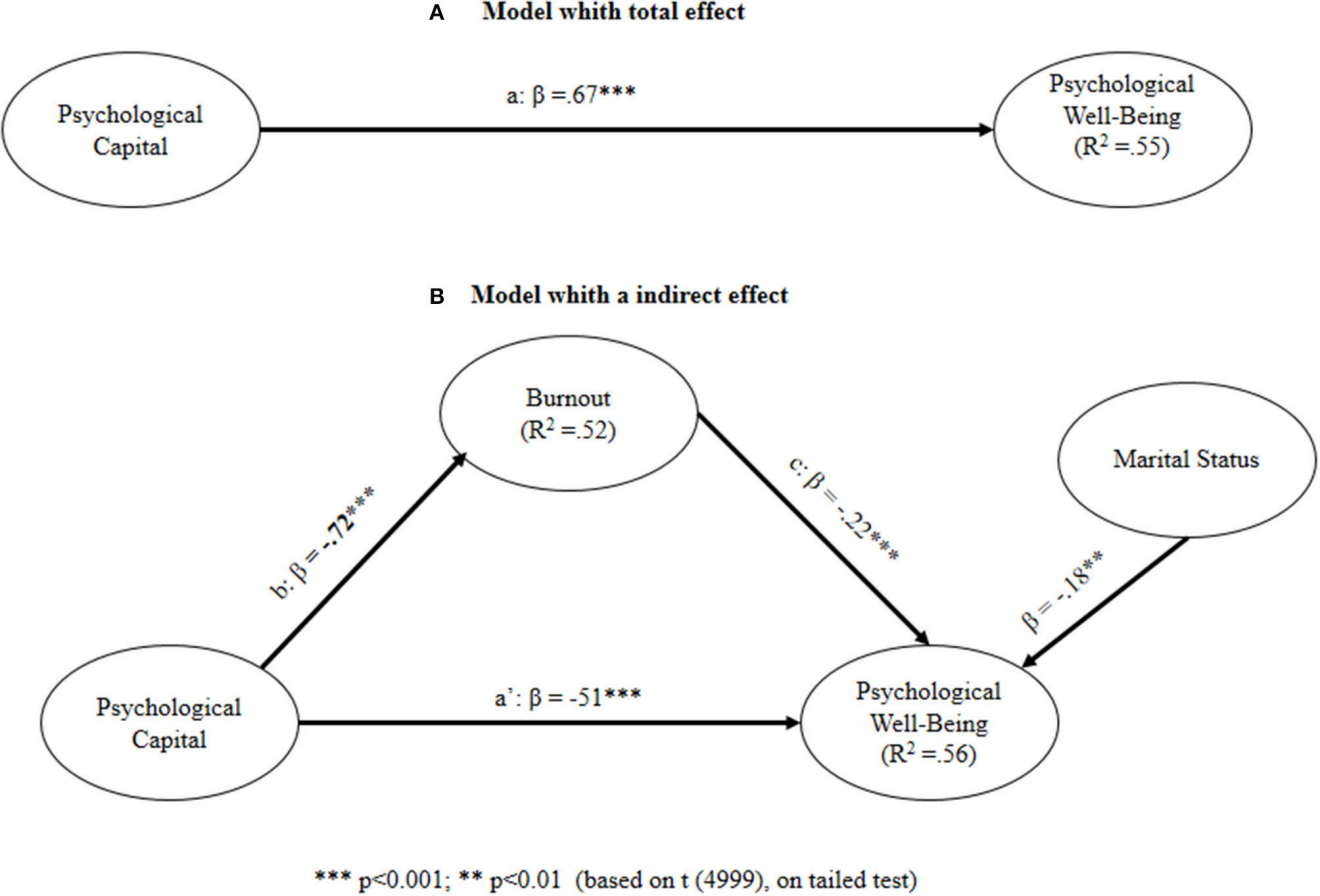
Path model analysis output **(A)** model whith total effect. **(B)** Model whith a indirect effect.^***^p < 0.001;^**^p < 0.01 [based on t (4999), on tailed test].

Table 4 and Figure 2 show that there is a direct and positive relationship between psychological capital (PC) and PWB. When we introduce burnout (B) as a mediator in the relationship between PC and PWB, the relationships between psychological capital (PC) and burnout (B), and burnout (B) and PWB, are significant. The total effect of PC on PWB (ß = 0.67) is the sum of the direct (ß = 0.51) and indirect (ß = −0.72^*^ß = −0.22 = 0.16) effect. In accordance with Shrout and Bolger (2002), if paths (PC→B) and (B→PWB) are significant and (PC→PWB) in step 2 (Model 2) is smaller than (PC→PWB) in step 1 (Model 1) by a non-trivial amount, we can say that there is an indirect effect of PC on PWB through B (mediator). According to Hair et al. (2014) we could say that burnout mediates the relation between psychological capital and PWB. It is a partial mediation, as the value of the Variance Accounted For (VAF) is 0.24 (0.16/0.67). The significance of the partial mediation role of burnout was examined with the bootstrapping method (bootstrap resamples = 5,000; Preacher and Hayes, 2008). Results of the bootstrapping method (standardized indirect effect = 0.16; 95% bias corrected confidence intervals = 0.02–0.32) support the significance of this partial mediation beyond the 0.05 level.

**Table 4:** Structural model results.

**Relationships**	**Step 1 (model 1)**	**Step 2 (model 2)**
		R^2^PWB = 0.56
	R^2^PWB = 0.55	R^2^B = 0.52
MS—> PWB	−0.20 (0.002)	−0.18 (0.003)
PC –> PWB	0.67 (0.000)	0.51 (0.000)
PC –> B		−0.72 (0.000)
B–> PWB		−0.22 (0.040)

*MS, Marital status; PWB, Psychological well-being; PC, Psychological capital; B, Burnout. p-values [based on t(4999), one-tailed test] concerning the standardized beta coefficients (β) are presented in parentheses*.

G^*^Power (Faul et al., 2007) showed a statistical power for R^2^ deviation from zero of 91.3 per cent, higher than the recommended level of 80 per cent. R^2^ for the dependent variables are higher than the cut-off level of 10 per cent and Q^2^ statistics tests (Q^2^ PC = 0.24; Q^2^ B = 0.27) obtained by blindfolding (Geisser, 1975) are also higher than zero, demonstrating the predictive significance of the model.

### Discussion and implications for practice

The study of psychological capital within an organization is a relatively new field, virtually non-existent in the field of direct support staff of specialist autism services. According to Salanova (2008), the need to create organizations that focus on workers' strengths to reach optimal organizational performance is becoming more evident. Positive Organizational Psychology argues that a worker's health is a goal in itself, and a legitimate objective that should be included in organizational policies. Stajkovic (2006) have shown the importance of personal strengths and psychological capacities, which can be measured, developed and managed in order to ensure an organization's best possible functionality and that its workers feel good at a psychological level. In this sense, this study provides empirical evidence to support our H1, and underlining the importance of enhancing the psychological capital of direct support staff of specialist autism services as a way to achieve higher levels of PWB.

Further understanding regarding how burnout mediates between the psychological capital and PWB of direct support staff of specialist autism services was achieved through examining a theorized path model. The results showed that fostering psychological capital (defined in terms of resilience, self-efficiency, optimism and hope) in a work environment should result in lower burnout which, in turn, should lead to higher degrees of PWB of direct support staff of specialist autism services. Our results do not allow us to assert, as proposed in H2, that burnout completely mediates the relationship between psychological capital and PWB. The data show that this is partial mediation. This means that psychological capital influences levels of PWB, both directly and indirectly (via burnout).

Our results support previous research in occupational health and psychological health that showed that PWB is impacted by the dimensions of psychological capital (Semmer et al., 2008; Hansen et al., 2015). Positive psychological capacities, the resources from which one can draw, seems are an important theoretical explanation of the mechanism by which such positive capacities impact on PWB (Avey et al., 2010). Findings from the study suggest that direct support staff of autism services who experience frequent negative emotions and infrequent positive emotions are more likely to draw on their internal positive psychological resources inherent in psychological capital (Sweetman and Luthans, 2010). As Wright et al. (2007) showed, our findings suggests that psychological capacities could be used to examine the differential effects of interventions that seek to foster PWB of the direct support staff of specialist autism services in the workplace Previous studies showed that psychological capital can be improved through training (Luthans et al., 2008). Such interventions must be designed while considering that psychological capital is a multidimensional construct which must be improved in each of its four dimensions.

Our results provide a way to integrate positive psychology with burnout. According to Farh et al. (2010), individuals try to protect themselves from stressful factors by increasing their positive resources. The findings from the study suggest, in line with the previous research (Gökhan Bitmişa and Ergenelib, 2015), that the positive psychological resources inherent in psychological capital of the direct support staff of specialist autism services may serve as a personal characteristic resource. Resource loss decreases motivation, and may eventually lead to burnout, whereas resource gain increases motivation and PWB. When the direct support staff of autism services experience negative feelings and states due to burnout as a result of their work tasks or environment, they draw on the positive psychological resources in psychological capital to counter the effects of burnout (Vazi et al., 2011). Our findings support those reported in previous research (Lizano and Mor, 2015; Harris et al., 2016), and emphasize the importance of strengthening the psychological capital of the direct support staff of specialist autism services, which will in turn help them improve their professional effectiveness, reduce feelings of emotional exhaustion and use cynicism as a coping mechanism less in their daily work. Many of the sources of burnout described by Maslach and Leiter (1997) could be reduced by increasing the psychological capital of the direct support staff of specialist autism services. People with high psychological capital do better in diffuse environments, handle change, adversities and risks better and learn from them. Direct support staff of autism services who have solid psychological capital learn how to overcome difficulties in their work environment and develop better coping mechanisms to deal with burnout (Manzano-García and Ayala Calvo, 2013), which will increase their levels of PWB. In conclusion, burnout has a partial mediating effect on the relationship between psychological capital and PWB. Direct support staff of autism services who show good psychological capital are less likely to suffer burnout and have greater PWB.

Our results provide encouraging guides for managers responsible for maintaining the PWB of direct support staff of autism services. Previous empirical studies have demonstrated that the psychological capital is state-like open to development and that it is possible to increase it by means of short training micro-interventions (Luthans et al., 2008, 2014). James et al. (2016) claim that training programmes increase job satisfaction and the feelings of well-being at work of the direct support staff of specialist autism services and this would in turn reduce staff sickness absence and staff turnover. Avey et al. (2010) believe that a minimal investment in psychological capital may be particularly useful for potentially enhancing the PWB of their workers. This investment would include workshops, individual and group therapy and support groups to enhance individuals' self-efficacy, optimism, hope and resilience. Strategies based on problem solving that allow the direct support staff of specialist autism services to strengthen and develop their own psychological capital and help reduce their levels of burnout need to be implemented. Training sessions should be developed throughout the year, and schedules should be based on the workers' availability. Our findings suggest that those responsible of the specialist autism services should focus on enhancing the PWB of its staff to ensure their own health. A “well-being office” to support and offer personalized monitoring of workers' strengths and weaknesses would be a significant step forward in this issue.

Marital status is the only variable included in the model as a control variable. Previous investigations have revealed that marital status is a source of social support which acts preventing and reducing the individual responses to stress and can predict PWB (Hank and Wagner, 2013). In a social environment, cohabitation with a partner functions as a safety net, making both partners develop a shared perspective in which they understand each other, help each other, accommodate each other, support each other and are connected to one another (Dwivedi et al., 2015). Our results, in line with those of Reneflot and Mamelund (2012), show a statistically significant relationship between marital status and PWB. And yet, the inclusion of the control variable in the model doesn't modify either the significance or the sign relations between psychological capital, burnout and PWB. At the same time, as was the case with previous studies, other sociodemographic variables were not significant in the explanation of psychological capital (Arum and Wustari, 2010; Caza et al., 2010), burnout (Lovell and Wetherell, 2016; Manzano-García and Ayala, 2017) or PWB (Benjak, 2011). Malekitabar et al. (2017) showed that gender does not have a moderating role in the relationship between psychological capital and burnout, nor in the relationship between PWB and burnout. Nevertheless, it is necessary to continue researching the possible moderating role played by sociodemographic variables in explaining psychological capital, burnout and PWB, and the relationship between them, since the results are currently inconclusive (Evans and Steptoe, 2002; Maslach and Leiter, 2008; Ding et al., 2015). The findings from our study may stem from the fact that the sample is fairly homogeneous in terms of gender (82% women), working hours per week (89% work 40 h or less), type of contract (77% have a fixed contract) and number of children (in 73% of cases, the number of children is below the average value).

### Limitations and future research

Like others, this research has several limitations that need to be addressed in future research. Firstly, this study used a relatively small-sized sample (*N* = 56). However, the sample size was sufficient to test the proposed model (Fornell and Larcker, 1981). Secondly, the causal direction of relationships between psychological capital, burnout and PWB remains unclear, given the use of a time lagged design. Adopting a time-lagged design we overcome many limitations associated with cross-sectional research and it let us to test the hypothesized relationship in a more rigorous manner (Valls et al., 2016). To make the direction of relationships between these variables clearer, longitudinal studies would be necessary. Thirdly, the different components of psychological capital could be related to different outcomes. Not all aspects of psychological capital have to be equally important for all jobs. Future research, conducted with different groups, could corroborate the validity of our results. Fourthly, in this study, we focused on psychological capital and burnout at an individual level. Some researchers (Avey et al., 2010) suggest the need to consider the contribution of group resources over and above personal resources. Meanwhile, Maslach and Leiter (2016) suggest that burnout should not be considered a syndrome of the individual, but should be considered as a characteristic of workgroups. Future research could consider these suggestions that could help improve our understanding of how burnout mediates the relationship between psychological capital and PWB of direct support staff of autism services. Finally, four self-reporting scales have been used to measure the model variables, which means that common method biases could influence our results. Although Spector (2006) has argued that this issue may be overstated, in order to minimize the potential impact of common method variance, since it has not been possible to obtain data from different sources, we have assessed the predictors and dependent variable at different moments in time. The amount of time that elapsed between the measurement of psychological capital, burnout and PWB was explicitly included in the design of the analysis to create a temporary, contextual and psychological separation.

## Author contributions

GM-G and J-CA have worked together and have made an active contribution to the conception, design, analysis, acquisition and interpretation of the data and drafting of the paper. GM-G and J-CA have both read the manuscript and have approved its definitive version.

### Conflict of interest statement

The authors declare that the research was conducted in the absence of any commercial or financial relationships that could be construed as a potential conflict of interest.
